# Implications of COVID-19 on the Teaching and Learning of Undergraduate Medical Imaging Students

**DOI:** 10.7759/cureus.32077

**Published:** 2022-11-30

**Authors:** Inayatullah S Sayed, Muhammad H Zamri

**Affiliations:** 1 Department of Diagnostic Imaging and Radiotherapy, Kulliyyah of Allied Health Sciences, International Islamic University Malaysia, Kuantan, MYS

**Keywords:** medical imaging students, clinical practice, impact on academic performance, online teaching and learning, covid-19

## Abstract

To preserve public health and prevent the spread of COVID-19, academic institutions curtailed face-to-face instruction and learning after the outbreak. The traditional techniques for education were modified, and new ways of instructing students were implemented. It presented a number of difficulties for the educational system, particularly for universities offering healthcare education. Therefore, the aim of this research was to look into how COVID-19 affected the teaching and learning of undergraduate medical imaging students.

The ScienceDirect, Oxford University Press Journals, Cambridge University Press Journals, and Taylor & Francis Online databases were searched, and a total of 14 papers met the inclusion and exclusion criteria and were selected for further analysis. The literature was analyzed using a thematic approach, with recurring themes brought to light.

The effects of COVID-19 on medical imaging education include but are not limited to the more rapid adoption of online education and new approaches to assessing and guiding students. Online teaching for medical imaging students influenced their learning environment, interaction, and motivation. The new COVID-19 safety requirements and procedures in hospitals have profoundly impacted clinical practice. Additionally, students’ research activities were also affected.

We anticipate that the findings of this study will enable us to be better equipped to assist students in comparable circumstances in the future.

## Introduction and background

Teaching is the fundamental engagement done by teachers, lecturers, and academic instructors alike for the learners, which are the students, to be able to achieve the desired understanding. It is a series of activities designed to enhance the internal learning process, according to Sequeira [1]. Its purpose is to actively include students in the process of learning by enabling their participation in the creation of knowledge. Teaching’s main goal is to help students transition from passively absorbing other people’s knowledge to actively creating their own and that of others. According to Christensen et al. [2], teaching is essentially about setting up the circumstances so that students may be in command of their own learning, both individually and collectively. Furthermore, one of the components of training healthcare professionals is that the instructor must be concerned about the well-being of his or her students [3]. Because stressed-out students don’t study as well, competent instructors make an effort to ensure that their students’ environment and basic living situations are satisfactory.

Sequeira [1] asserts that learning is a change brought about by acquiring a new ability, comprehending a scientific rule, and altering one’s perspective. It is the action or process of learning something new via study, practice, instruction, or experience. According to Prozesky [3], when learning, we acquire abilities and attitudes in addition to information and facts. Since their daily job has an effect on the health of the people they serve, health professionals need to be particularly aware of this. Information, skills, and attitudes are acquired in a variety of ways. For example, a new idea can be learned through discussion, but skills are acquired by practicing and receiving feedback.

Students in healthcare courses participate in a variety of activities including evaluations, research, and clinical practice designed to enhance their learning experience. Assessments provide evidence that students are acquiring knowledge and skills. It also measures the effectiveness of the delivery of teaching and learning strategies by the students. Aside from assessments, research is also included as a form of learning. Numerous curricula in many higher education institutes require their students to participate in research projects as a requirement in their senior year courses because of the importance and benefits they bring to the teaching and learning process. The research processes themselves have a beneficial impact on learning objectives as undergraduates prepare for their respective professions [4]. Additionally, Framtz and Rhoda [5] highlighted that education is given in both classroom and clinical settings in allied health courses. Students learn more about their professional responsibilities through clinical practice and placements, which help connect theory and practice.

The severe acute respiratory syndrome coronavirus 2 (SARS-CoV-2) was found in people in Wuhan, Hubei Province, China, in December 2019, during an outbreak of pneumonia. The COVID-19 pandemic caused a myriad of effects on the daily lives of people, ranging from people’s lifestyles to socialization, to the global economy [6]. The World Health Organization designated COVID-19 as a public health emergency of worldwide concern from the first outbreak on January 30, 2020. Public health measures were taken in an attempt to slow the transmission and spread of COVID-19. These measures are important to make sure the healthcare system is not overburdened and is able to protect its workforce. One of those recommended measures is the use of physical distance, cancellation of mass gatherings, and closure of academic institutions [7]. It caused multiple challenges for education, especially at universities offering healthcare education, and made it difficult to create an effective learning environment.

In courses and practical sessions, COVID-19 changed teaching strategies and introduced new approaches to the teaching and learning process. The COVID-19 fighting measures unintentionally made practical and hands-on lectures difficult. This includes switching the teaching and learning process, assessments, group work, presentations, and evaluations to an online platform. This also includes the teaching and learning process for medical imaging students. Examples include the quick transition from the traditional model of in-person instruction to online learning and the effect on clinical placements [8]. The clinical programs of many students have had to be canceled or postponed at medical imaging training facilities worldwide. Another effect of COVID-19 on teaching and learning in a clinical placement context, according to a survey done for a study by Rainford et al. [8], is the scarcity of patients for practice and evaluation. There were also problems with staff who were sick or staying home out of fear of spreading the disease to family and friends and out of worry for their own safety.

Nevertheless, online learning promotes student-centered learning as it provides comfort for some and uses self-directed and asynchronous learning. Prerecorded lectures made it easy for students to go back and get through the whole lecture at their own pace [9]. It can be seen that the pandemic provided some advantages to the students. The effects of COVID-19 on medical imaging students’ instruction and learning must be further investigated.

This is done as a means to limit the spread of COVID-19 in their respective countries. Online distant learning was regarded at the time as the most effective method for ensuring continuity in the teaching and learning process. It is known to be flexible, accessible, and effective, but with certain drawbacks.

The study by Chung et al. [10] mentioned that such drawbacks include the lack of human interaction to sense the students’ comprehension via facial expressions and body language. These disadvantages, without a doubt, pose obstacles to the successful delivery of teaching and learning. In the survey conducted by Chung et al. [10], degree students who mostly undergo online learning methods in East Malaysia agreed that internet connectivity was the number one challenge. Due to a lack of face-to-face contact, 68% of students lack motivation. Similar studies have been conducted to explore whether COVID-19 directly or indirectly affects the teaching and learning of students. However, few studies have been reported on medical imaging students.

This research aims to shed light on the full picture, including the pros and cons, of online education to better address the difficulties students confront. The results from this study could aid academic institutions in shaping and planning remote teaching and learning. Future teaching and learning could be improved, especially during an ongoing pandemic or if the education system faces a similar situation where remote teaching and learning must be implemented, and more students would be less negatively affected.

## Review

Methods

Search for Relevant Articles

A literature review was undertaken as part of this study. A literature review is a retrospective and reflective analysis of prior research on a particular subject that may be used for a number of purposes [11]. It serves to contextualize and inform further research through summaries and critical analysis. In the end, a literature review’s conclusion, which is based on research findings, can give practitioners and policymakers insight and help them make decisions.

The goal of this study was to collect as much literature as possible from available databases related to the study’s research topic. The topic that was chosen is the impact of COVID-19 on the teaching and learning of undergraduate medical imaging students. When searching databases, the literature was searched using keywords from available databases related to the study’s research topic. The keywords were selected from the research questions and objectives. In this case, the search terms were “impact,” “COVID-19,” and “medical imaging students.” The term “medical imaging students” is broadened as different institutes use different terms but with similar meanings, such as “radiography students,” “medical imaging undergraduates,” and “student radiology technologists.” This is done to ensure the corresponding literature is included in the search.

The search terms were then combined to construct a search statement using Boolean operators AND, OR, and NOT to further improve search results. The AND was used when searching for literature that contains both words in that literature. This is to narrow the search further and be more specific. The OR was used to search for literature containing either one word or another and is useful to broaden the search. Meanwhile, NOT was used for one term but not another. This is used to exclude irrelevant results and reduce false-positive results [12].

Electronic databases were extensively used for the literature search as they were the most readily accessible source. Most of the databases used are ScienceDirect, Oxford University Press Journals, Cambridge University Press Journals, and Taylor & Francis Online. ScienceDirect is one of the largest online databases that covers scholarly literature from almost any discipline. Access to a sizeable database of academic and medical research is available through ScienceDirect. The greatest electronic collection of full-text and bibliographic material on science, technology, and medicine is also contained there. However, databases that provide open access to literature are also used in the literature search. Another technique used to improve the literature search is snowballing. Snowballing is tracking down references or citations using key literature as a starting point to find other relevant titles on this subject [13].

Criteria for the Selection of Articles

The selection process of articles was done using the inclusion and exclusion criteria. These criteria were developed based on the objectives of this research. Due to the search engine’s word pick-up, the items frequently look related but are actually deceptive. The papers were then screened for key information needed for data extraction using the inclusion and exclusion criteria [12]. The criteria should not be too narrow, as this may result in studies being missed, or too broad, as this will make data analysis difficult and time-consuming. The inclusion and exclusion criteria for the selection of articles in this study are listed in Table 1.

**Table 1:** Inclusion and exclusion criteria for the selection of articles

Inclusion criteria	Exclusion criteria
The time period of the articles must be between the years 2020 and mid-2022.	The time period of the articles is before 2020.
The study involved medical imaging/radiography students (undergraduates).	The study involved students of other courses, postgraduates, and PhD students.
The study relates to the COVID-19 pandemic.	The study relates to other issues not related to COVID-19.
The study involves the teaching and learning of medical imaging students, including clinical training, classes, and examinations pre-pandemic.	The study involves teaching and learning of medical imaging students before the COVID-19 pandemic began.
The study design of articles includes either qualitative and quantitative study or both.	Articles are written in languages other than English.
Articles are written in English.	

The literature obtained from the database search was reviewed by its title and abstract, and the selected studies were only reviewed if they satisfied the inclusion criteria. Studies that were found irrelevant and did not satisfy the inclusion criteria were rejected. The searches were narrowed down only to studies between 2020 and mid-2022, as the COVID-19 pandemic only emerged in 2020 and the impact on medical imaging students was only recorded and evaluated during that time. The study includes medical imaging students, specifically undergraduates. However, there should be some aspects that were included, as it not only includes medical students but also undergraduate and healthcare students. The study excludes students outside of healthcare courses, postgraduates, and PhD students. Furthermore, the study includes quantitative and qualitative articles, and articles in English were selected due to time constraints on waiting for translations.

Article Extraction and Organization

The articles obtained from the selection were read carefully and understood thoroughly. This was done to avoid being incomplete or inaccurate. The reading was also done actively and critically. Information applicable to the study was extracted from each selected article in line with the objectives of this research and was able to answer the research question. Prior to this procedure, the retrieved studies were arranged separately, with information about each study recorded in a table or spreadsheet. The initial table consists of the authors’ family names, year of publication, a brief summary of the study, methodology, results, and a simple interpretation of the results. The goal of this method was to cut down on the time it took for the reviewer to process and write down data by focusing only on the most important parts of the study instead of spending time getting rid of useless and irrelevant data.

Data derived from the extraction process were then tabulated before performing the summary and synthesis. Subsequently, the categorization of information depends on the findings of those studies. In terms of their conclusions and methodologies, research should be compared and contrasted to highlight commonalities and discrepancies. The analysis should represent all findings, not just those that support one’s position. The studies were then analyzed, and emerging themes were identified and summarized.

Results

Selection of Articles

**Figure 1:**
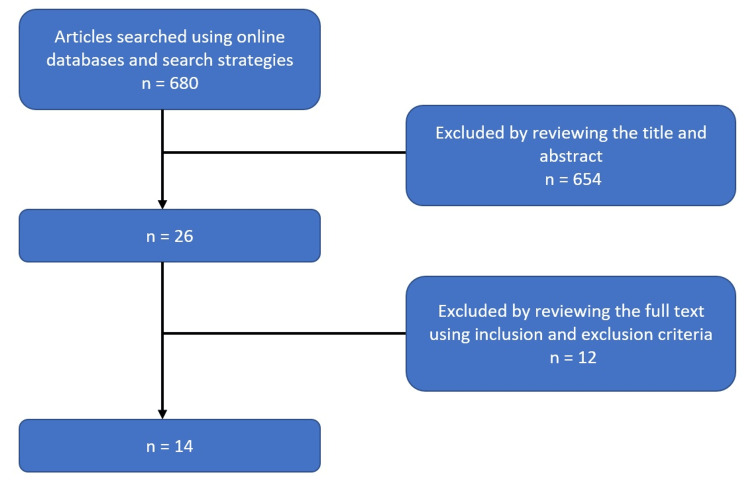
Flow diagram of the selection process of articles

The initial search using online databases, mainly ScienceDirect, Oxford University Press Journals, Cambridge University Press Journals, and Taylor & Francis Online, returned a total of 680 articles. Advanced search strategies were done on the Cambridge University Press Journals online database to further narrow down the search results, such as changing the term “medical imaging” to “radiography.” Following that, the titles and abstracts were reviewed. There were still 26 potentially pertinent articles. The full papers were then downloaded and checked against the inclusion and exclusion criteria for relevance to the research’s title and research question. Duplicate studies were removed. The 14 remaining articles that satisfied the inclusion criteria were then evaluated. The article search and selection flow diagram is shown in Figure 1.

Fourteen articles that were chosen as shown in Table 2 included a variety of study approaches, including the following: nine quantitative surveys and one in-depth interview, two commentaries, one editorial, and one literature review. The participants of the studies consist of medical imaging students, health science students, medical sciences (with medical imaging students included), radiology, and medical imaging educators, including clinical instructors. Data analysis revealed several recurring themes, including evaluations and instructional procedures, accessibility, clinical practice, learning environment, simulations and laboratories, research, rapid transition to online learning, and stress and anxiety. Table 2 presents a summary of each selected article’s description from this review and the articles’ conclusions.

**Table 2:** Summary of the selected articles

Author(s) and publication year	Title	Type of study	Country	Theme(s)
Rainford et al., 2020 [8]	The impact of COVID-19 upon student radiographers and clinical training	Survey	International	Assessments and learning process, clinical practice
Chung et al., 2020 [10]	Online learning readiness among university students in Malaysia amidst Covid-19	Survey	Malaysia	Learning environment, interaction, and motivation, assessment and learning process
Gumede and Badriparsad, 2022 [14]	Online teaching and learning through the students’ eyes - uncertainty through the COVID-19 lockdown: a qualitative case study in Gauteng province, South Africa	Survey	Gauteng, South Africa	Assessments and learning process, accessibility, rapid transition to online learning, learning environment, interaction, and motivation
Teo et al., 2020 [15]	Coping with COVID-19: perspectives of student radiographers	Commentary	Singapore	Rapid transition to online learning, assessments and learning process, learning environment, interaction, and motivation, clinical practice, stress and anxiety
Astirbadi and Lockwood, 2022 [16]	COVID-19: a literature review of the impact on diagnostic radiography students	Literature review	United Kingdom	Assessments and learning process, rapid transition to online learning, clinical practice
Ofori-Manteaw et al., 2022 [17]	Impact of the Covid-19 pandemic on clinical radiography education: perspective of students and educators from a low resource setting	Survey	Ghana	Rapid transition to online learning, clinical practice
Chandrasiri and Weerakoon, 2022 [18]	Online learning during the COVID-19 pandemic: perceptions of allied health sciences undergraduates	Survey	Sri Lanka	Rapid transition to online learning, accessibility, learning environment, interaction, and motivation
Elsalem et al., 2021 [19]	Remote E-exams during Covid-19 pandemic: a cross-sectional study of students’ preferences and academic dishonesty in faculties of medical sciences	Survey	Jordan	Rapid transition to online learning, assessments and learning process
Elshami et al., 2021 [20]	Satisfaction with online learning in the new normal: perspective of students and faculty at medical and health sciences colleges	Survey	United Arab Emirates	Rapid transition to online learning
Alhasan and Al-Horani, 2021 [21]	Students’ perspective on the online delivery of radiography & medical imaging program during COVID-19 pandemic	Survey	United Arab Emirates	Assessment and learning process, rapid transition to online learning
Lawson Jones et al., 2021 [22]	The experience of diagnostic radiography students during the early stages of the COVID-19 pandemic - a cross-sectional study	Survey	United Kingdom	Rapid transition to online learning, accessibility
Tay et al., 2020 [23]	Clinical placements for undergraduate diagnostic radiography students amidst the COVID-19 pandemic in Singapore: preparation, challenges, and strategies for safe resumption	Survey	Singapore	Assessments and learning process
Tay et al., 2021 [24]	The needs and concerns of clinical educators in radiography education in the face of COVID-19 pandemic	Commentary	Singapore	Clinical practice, assessments and learning process
Currie et al., 2020 [25]	COVID-19 impact on undergraduate teaching: medical radiation science teaching team experience	Editorial	New South Wales, Australia	Learning environment, interaction, and motivation, assessments and learning process, research

Emerged Themes

Rapid transition from classroom to online teaching and learning: According to the literature review, COVID-19 has an impact on medical imaging students’ teaching and learning because of the quick switch to online instruction. Nine of the 14 studies [14-22] have covered this topic in their discussions. It was concluded by the participants in the study of Gumede and Badriparsad [14] that face-to-face instruction is superior to distance education. It is mentioned that some students have difficulty concentrating during online lectures because they are unfamiliar with learning through their gadgets. In addition, Teo et al. [15] noted that it was challenging to make the shift from a class that adhered to a set schedule to one that allowed for greater flexibility and was conducted at home.

According to Ofori-Manteaw et al. [17], 44% of students thought their online learning experiences were fair, while 25.2% said they were poor or extremely poor. Moreover, the data reveals that just 27.5% of the students surveyed consider themselves to be technologically savvy, whereas all of the instructors in this study were experienced with online platforms. In a separate research performed by Elshami et al. [20], there is less satisfaction with the abrupt switch to online delivery of the curriculum since there was not enough time for preparation in addition to the demanding working circumstances caused by the pandemic. Additionally, Lawson Jones et al. [22] discussed that individuals with less technical literacy may face difficulties.

Assessment and learning process: COVID-19 impacted the teaching and learning of medical imaging students in terms of assessments and learning processes. It was found by Tay et al. [23] in their survey results that 51% and 54% of year 3 and year 2 students, respectively, were worried about achieving the learning goals owing to the low quantity and variety of cases faced in clinical practice. The amount and diversity of cases accessible to students for their case report assignments have decreased, which is consistent with the conclusion reported by Teo et al. [15].

In their study, Chandrasiri and Weerakoon [18] found that 59.7% of respondents believed that online learning is more user-friendly than conventional learning. In addition, 62.6% of respondents stated that online learning was convenient and permitted students to learn at their own pace. This study also indicated that delivering courses through online learning requires less time.

There has been a change in the assessment methods used because of the restraints in clinical settings due to COVID-19. According to Tay et al. [24], the prior summative clinical tests that were carried out by an academic institution’s designated examiner were changed to be replaced with several formative evaluations that were spread out during the clinical placement. Correspondingly, in the study by Currie et al. [25], this situation may be an opportunity to create more authentic teaching, learning, and assessments designed to draw out student understanding.

Online tests were then administered and regarded as the main form of evaluation since online teaching and learning were adopted as a strategy during the COVID-19 pandemic. According to Elsalem et al. [26], 49.86% of respondents stated that more time and effort were required to prepare for online examinations.

Accessibility: COVID-19 impacted the teaching and learning of medical imaging students in terms of accessibility, whether or not the students had consistent access to the lectures and study materials. According to a study done by Gumede and Badriparsad [14], the reasons why students are unable to view the majority of online courses include connection challenges and the costs associated with using data. Students who have limited or no access to internet resources may be subject to prejudice, as highlighted by Chandrasiri and Weerakoon [18]. Similarly, in the study conducted by Lawson Jones et al. [22], students who do not have constant access to the internet or devices may find it difficult to engage.

Clinical practice: The study conducted by Tay et al. [23] discussed that the students were unable to put theories into practice. Students’ clinical assignments have been cut short, limiting their exposure to the field of radiography in practice. The lack of cases also contributes to the lesser hands-on experience. Tay et al. [24] also reported similar situations where the duration, movement, and rotation of clinical placements were reduced.

Of the respondents, 91% believed that the pandemic had influenced students’ clinical placements, according to the research by Ofori-Manteaw et al. [17]. The study discussed the issue of students’ inability to be put in clinical settings appropriately. Students who were able to undergo clinical practice, however, experienced less supervision by the hospital staff because of the increase in workload. The staff could not properly teach the students during this period. Furthermore, for international students, the disruption of clinical training reduces their opportunities to experience additional competencies in a foreign healthcare system.

Learning environment, interaction, and motivation: According to Gumede and Badriparsad [14], some participants were at home during the lockdown period, where the environment was not suitable for learning. In addition, the participants lack the desire to engage in online learning due to a lack of sufficient resources and inadequate computer abilities. Furthermore, Teo et al. [15] discussed that not all students have a conducive environment for home-based learning. This study also mentioned that the pacing required for the students differed and that each student absorbed online content differently. Although given the same source material, there is now a divergence in students’ understanding of the lectures. However, Currie et al. [25] found that online platforms for live lectures had better attendance than face-to-face lectures, despite the fact that the students’ engagement levels were lower.

Research: According to Currie et al. [25], researchers have stopped doing qualitative in-person interviews for their studies. The additional effort caused a delay in the process of conducting research, and as a result, a number of approvals were ended early with COVID-19 serving as the finish point for some of them. This is reinforced by the study Ofori-Manteaw et al. [17], which found that students in year 4 encountered difficulties in data collecting and lacked supervision from their supervisors when the pandemic was in effect.

Discussion

Effects of Rapid Transition From Classroom to Online on Teaching and Learning

It was discovered that COVID-19 has an effect on the teaching and learning experiences of medical imaging students since it was determined to be indirectly responsible for the quick shift to online learning. Participants in the study conducted by Gumede and Badriparsad [14] reported that they felt contact learning to be more useful than online teaching and learning since all of the students were able to participate. Changes in the learning pattern are one of the subthemes of this study’s analysis. According to the results of this study, students spend a considerable amount of time just getting acclimated to using their devices for online learning. This is consistent with the findings of Ofori-Manteaw et al. [17], which showed that making the switch to online teaching and learning takes a lot of planning, consideration, and effort. Some students lack access to or are unfamiliar with the resources needed to facilitate online learning. Individuals who are less technically literate might also have difficulties [22]. Educators, unlike students, had already been exposed to online education delivery. Because of the unexpected spread of the COVID-19 pandemic, educational institutions might not have had time to prepare their students for the shift to online education. Furthermore, comparable explanations are given for the drop in satisfaction that was linked to the introduction of online course delivery as mentioned by Elshami et al. [20]. When asked about the length of time it took to access course materials, one-third of the students expressed dissatisfaction.

According to Teo et al. [15], it was challenging to switch from a fixed, timetable-based class to flexible, at-home learning. Lectures and slides are now prerecorded and posted for self-study; therefore, there is no longer a strict schedule to follow. The students in this study explained that adequate pacing is required to avoid burnout from studying too much, too fast, or becoming overwhelmed.

Implications on Assessment and Learning Process

COVID-19 impacted the teaching and learning of medical imaging students in terms of assessments and learning processes. Due to the few and different kinds of cases seen in clinical practice, Tay et al. [23] found that 51% and 54% of year 3 and year 2 students, respectively, were worried about completing the learning objectives. It should also be noted that students were further excluded from having placements in high-risk areas, further lowering the number of encounters. This conclusion is comparable to that of the study by Teo et al. [15], which showed a drop in the variety and quantity of cases that students can choose from, which they can draw material for case study reports.

In their study, Chandrasiri and Weerakoon [18] found that 59.7% of respondents concurred that online learning is more user-friendly than conventional learning. Also, 62.6% of respondents said that studying remotely was more flexible and gave them more control over their education. This study also found that online learning takes less time to deliver courses. This study showed a different perception of online teaching and learning when compared to other studies included in this review.

Assessment methods had been modified because of the restraints in clinical settings due to COVID-19. Previous summative clinical tests conducted by an academic institution’s designated examiner were replaced with many formative evaluations throughout the clinical placement [23]. Correspondingly, this situation may provide an opportunity to create more authentic teaching, learning, and assessments designed to elicit student comprehension [25]. In light of online examinations, to prevent cheating, assessments would be made in a way where the answers to the examinations need to delve into students’ conceptualization of key ideas to help them synthesize new information or solve difficulties. Subsequently, due to the fact that the COVID-19 pandemic saw a rise in the usage of online education, online examinations were conducted and are considered a primary mode of assessment. According to Elsalem et al. [26], 49.86% of respondents stated that more time and effort were required to prepare for online examinations. The variety of instructional strategies, study aids, and resources utilized in online teaching and learning may be responsible for the extra work. For instance, asynchronous learning makes use of slides, recorded movies, handouts in PDF format, and narrated PowerPoint presentations. As a result, students may have to put in more effort studying for online examinations from multiple sources.

Impact on Accessibility

COVID-19 impacted the teaching and learning of medical imaging students in terms of accessibility, whether or not the students had consistent access to the lectures and study materials. According to the research by Gumede and Badriparsad [14], students report problems with internet connectivity and data as the main reasons they are unable to view most online courses. In this study, it was discussed that online teaching and learning do not consider the students from rural areas that have internet connectivity issues. This is coupled with issues with incompatible devices. Findings from this research support the idea that recording lectures should be an option for those who are unable to participate in online lectures. When compared to traditional classroom settings, where all students have equal access to materials, Chandrasiri and Weerakoon [18] argued that students with less or no access to digital resources might be at a disadvantage. This is likely as the accessibility of the internet is low in countries such as Sri Lanka, making the gap more prevalent.

Effects on Clinical Practice

Since medical imaging is a hands-on field, the lack of practical training and the reduction in clinical rotation hours have had a major influence on teaching and learning. Tay et al. [23] discussed that the students were unable to put theories into practice. Because the period of the students’ clinical internships has been shortened, they have less time to spend in the practical environment of radiography, and as a result, they have less experience working in the field. In healthcare institutions, new temporary rules were put in place to limit the number of placements. That resulted in yet another adjustment to the structure of clinical placement: a reduction in the duration of each placement by half.

Students also reported less acceptance of students by healthcare facilities and less supervision by hospital staff and clinical educators. Precautions and safety measures were implemented to reduce congestion in radiology departments’ activities. This may be due to a number of factors, including the need to enforce social/physical distance and safety procedures, the upkeep of personal protection equipment, and the possibility of transmission of COVID-19. In addition, because of the extra work, hospital staff may also have less time to teach and supervise students.

Impact on Learning Environment, Interaction, and Motivation

The learning environment, interaction, and motivation of medical imaging students have been affected by the COVID-19 pandemic. The study by Gumede and Badriparsad [14] reported that some of the participants were at home during the lockdown period, where the environment was not suitable for learning. Students have a lack of motivation toward learning online as a result of the shortage of necessary resources and their lack of computer skills. No face-to-face conversations make it difficult to consult lecturers about their study difficulties, as there is immediacy in the conversation flow in face-to-face consultations, which could lead to better linkages between theories taught, leading to a better understanding of the study material [15]. Emailing lecturers back and forth is difficult, as sometimes loss of communication occurs.

Students’ efforts to learn were hindered by many domestic disruptions. With face-to-face learning, students could easily engage in small-group discussions and bounce ideas off each other. This allows for constructive engagement with classmates and peers. With online learning, students will study at a different pace and consume online content differently. Although given the same source material, there is now a divergence in students’ understanding of the lectures. Group discussions with classmates and peers became more difficult; with challenges with internet connections, online group discussion yields less output than desired.

However, Currie et al. [25] found that although live lectures delivered via online platforms had better attendance than face-to-face lectures, students in live lectures were less engaged personally. Students who are highly involved in their in-person classrooms also maintain that level of engagement in their online learning environments. Students’ involvement levels have been shown to be lower as a direct consequence of the greater reported attendance in live lectures using online platforms as opposed to traditional face-to-face sessions. Even if the students get the impression that there is more involvement, the problem resides in the type of engagement. Students would rather ask questions through the “chat” feature than unmute their microphones and ask inquiries directly; as a result, they are more involved technologically but less engaged personally.

Implications on Research

Students enrolled in medical imaging programs were influenced by the COVID-19 outbreak since it limits the human interaction research that can be conducted. Researchers have reportedly given up on doing in-person qualitative interviews, as stated by Currie et al. [25]. After it became clear that obtaining the requisite ethical permits would be a time-consuming and arduous procedure, interviews were finally moved to be conducted online. In addition, there was a decrease in the number of people participating in the study, and some research projects were abandoned because of connectivity problems. Studies that were sensitive to time constraints were the ones that experienced the consequences first and foremost, e.g., research, analysis, and synthesis assignments for undergraduate students [25]. A number of current experiments were significantly hampered by the COVID-19 pandemic, which prompted reevaluation and the possibility of continuing with some of the investigations. Some experiments were altered, but rather than utilizing real people as participants, the investigations relied on nonhuman interaction.

## Conclusions

COVID-19 affected medical imaging students’ learning in multiple ways. Due to the unexpected move to online distant education, students had trouble focusing during lectures. Students were not familiar with online learning tools, unlike teachers. COVID-19 changed summative to formative evaluations, which influenced assessments and learning. Assessments were designed to measure student knowledge, not just responses. Online examinations prompted worries about academic integrity. New safety rules and procedures to avoid COVID-19 in hospitals have impacted clinical practice. COVID-19 influenced medical imaging students’ learning environment, interaction, and motivation because of online distant teaching and learning. This altered classmate, student, and lecturer interactions. The research activities of students were affected in terms of progression, validity, and data collection. The outcomes of this study ought to make it easier for us to assist students in similar circumstances in the future.
